# Estradiol-Induced Potentiation of Dopamine Release in Dorsal Striatum Following Amphetamine Administration Requires Estradiol Receptors and mGlu5

**DOI:** 10.1523/ENEURO.0446-18.2019

**Published:** 2019-02-13

**Authors:** Zhimin Song, Hongyan Yang, Elizabeth M. Peckham, Jill B. Becker

**Affiliations:** 1 Molecular and Behavioral Neuroscience Institute; 2 Department of Psychology, University of Michigan, Ann Arbor, Michigan 48109; 3 Department of Psychiatry and Biobehavioral Science, Semel Institute for Neuroscience and Human Behavior, and Hatos Center for Neuropharmacology, University of California, Los Angeles, California 90095; 4 Department of Biology, Concordia University, Ann Arbor, Michigan 48105

**Keywords:** addiction, drug abuse, gonadal hormones, reward, sex difference

## Abstract

Estradiol potentiates behavioral sensitization to cocaine as well as self-administration of cocaine and other drugs of abuse in female rodents. Furthermore, stimulated dopamine (DA) in the dorsolateral striatum (DLS) is rapidly enhanced by estradiol, and it is hypothesized that this enhanced DA release mediates the more rapid escalation of drug taking seen in females, compared with males. The mechanisms mediating the effect of estradiol to enhance stimulated DA release were investigated in this study. Using *in vivo* microdialysis and high performance liquid chromatography coupled with electrochemical detection, we first examined the effect of estradiol on amphetamine-induced DA increase in the DLS of ovariectomized rats. We then tested whether the potentiation of this DA increase could be blocked by the estradiol receptor antagonist, ICI 182,780 (ICI), or an antagonist to the metabotropic glutamate receptor subtype 5 (mGlu5), 2-methyl-6-(phenylethynyl)pyridine (MPEP). There is evidence that estradiol receptors collaborate with mGlu5 within caveoli in DLS and mGlu5 is hypothesized to mediate many of the effects of estradiol in the addiction processes in females. Our data show that estradiol enhances the DA response to amphetamine. Either ICI or MPEP prevented the effect of estradiol to enhance DA release. Importantly, our results also showed that neither ICI or MPEP alone is able to influence the DA response to amphetamine when estradiol is not administrated, suggesting that ICI and MPEP act via estradiol receptors. Together, our findings demonstrate that estradiol potentiates amphetamine-stimulated DA release in the DLS and this effect requires both estradiol receptors and mGlu5.

## Significance Statement

The present study provides important information on the neurobiological mechanisms underlying the exacerbating effects of E2 on addictive behavior by showing blockage of E2 receptors or mGlu5 reduces E2-induced potentiation of DA release in the rat striatum following by amphetamine injections. Our data suggest that targeting E2 receptors or mGluRs could have treatment potentials for E2-related disorders in areas such as, but not limited to, drug addiction.

## Introduction

Women are more susceptible to drugs of abuse than men. They escalate faster from initial use to addiction, take more drugs when addicted, and have a harder time staying abstinent (Bobzean et al., 2014). This is mirrored in animal models, female rats acquire drug self-administration at a faster rate, are more motivated to take drugs, and respond stronger to drug cues during reinstatement (Becker 2016; Song et al., 2018).

It is suggested that these sex differences are regulated at least in part by estradiol (E2). Indeed, there is considerable evidence that shows the potentiating roles of E2 in cocaine self-administration, cocaine behavioral sensitization, and dopamine (DA) signaling in the nucleus accumbens (NAc) following cocaine administration (Hu and Becker, 2008). Despite this mounting evidence, how E2 enhances stimulated DA release or addiction-related behaviors are less well understood.

Many of the E2 effects involve intracellular estrogen receptors ERαs and ERβs (Foster 2012; Borrow and Handa, 2017). Recently, E2 was also shown to bind to a membrane G-protein-coupled receptor GPER-1 (Long et al., 2014). Depending on the types/locations of the receptors, the effects of E2 can range from minutes (non-genomic effects) to days (genomic effects; Ervin et al., 2015). In dorsal striatum (DS), a region that is critical for habitual drug taking behavior, E2 modulates behavior by acting on GABA medium spiny neurons (MSNs; Mermelstein et al., 1996) and by altering DA transmission indirectly through a presynaptic mechanism (Xiao and Becker, 1998; Schultz et al., 2009).

In the present study by using *in vivo* microdialysis and high-performance liquid chromatography (HPLC) coupled with electrochemical detection (ECD), we first examined effects of E2 on amphetamine (AMPH)-induced DA elevation in the striatum of female rats. We then tested whether the observed potentiated DA elevation could be blocked by an E2 receptor antagonist ICI 182 780 (ICI) or an antagonist to the metabotropic glutamate receptor subtype 5 (mGlu5), 2-methyl-6-(phenylethynyl)pyridine (MPEP) in the striatum as there is evidence that mGlu5 is required for many of the effects of E2 in addiction processes (Martinez et al., 2014, 2016).

## Materials and Methods

### Animals

Female Sprague-Dawley rats (weighting 200–225 g at the beginning of each experiment; were obtained from Harlan Laboratories or Charles River Laboratories) and housed in groups of two or three per cage before cannula implantation and singly housed after cannula implantation, under a 14/10 h light/dark cycle. The rats were housed in a room maintained at a constant temperature of 20–21°C, with phytoestrogen-free rodent chow (2014 Teklad Global, 14% protein rodent maintenance diet, Harlan rat chow; Harlan Teklad) and water available *ad libitum*. All procedures were performed according to the protocol approved by the Committee for Use and Care of Animals at the University and were in accordance with the NIH *Guide for the Care and Use of Laboratory Animals*.

### Ovariectomy

Approximately 1 week after arrival, all animals underwent bilateral ovariectomy (OVX). The OVXs were conducted using a dorsal approach under anesthesia of ∼2% isoflurane/oxygen. The skin was opened with an incision ∼1 cm long along the midline just below the ribs, and a small incision ∼0.5 cm long was made through the muscle 1.5–2 cm lateral to the midline. The ovary was externalized with blunt forceps, and the tissue between the ovary and uterus was clamped with a hemostat. The ovary was removed, and the hemostat remained in place until there was no bleeding before being released. The uterus with associated tissue was then returned to the abdomen. The procedure was repeated on the other side, and the wound was closed with 9 mm wound clips. The wound clips were removed after 14 d of OVX. After 7 d of recovery, all animals underwent vaginal lavage testing daily for 10 consecutive days to confirm cessation of cycling.

### Cannula implantation

Two to 3 weeks after OVX, all rats received buprenorphine (0.01 mg/kg, s.c.) or carprofen (5 mg/kg, s.c.) 30–60 min ahead of the cannula implantation surgery. During the surgery, all rats were anesthetized with ketamine (60 mg/kg, i.p.) and dexmedetomidine (0.3 mg/kg, i.p.). Guide cannulae (matching for CMA/11 probes, CMA/Microdialysis, or MAB 6 probes, SciPro; 4 mm membrane length) were inserted through the skull aimed at the striatum (AP +0.20 mm, ML ±3.00 mm, DV −1.50 mm) using standard stereotaxic techniques. The cannulae were held in place with acrylic polymer (Lang), which was secured to the brain with three to four stainless steel jewelry screws (Small Parts). A solid stylet was placed in each cannula when not in use, to keep the cannula patent. Animals were allowed to recover for at least 5 d before microdialysis. Starting 1 d after the surgery (both cannula implantation and OVX), rats were administered with carprofen (5 mg/kg, s.c.) daily for 3 consecutive days and triple antibiotic was given when necessary on observation. All rats were observed at least once daily for 10 consecutive days to ensure their recovery.

### Preparation for microdialysis

Animals were anesthetized with 3% isoflurane and maintained with 2% isoflurane during the procedure of removing the stylet and inserting a microdialysis probe into the brain through the guide cannula. Probes were placed into the brain 12–18 h in advance of the testing to allow sufficient time for the injury-related release associated with probe implantation to subside. Animals were placed in the test chamber (31 × 25 × 25 cm) with continuous white noise. The microdialysis probes were attached to syringes mounted on the syringe pump, and a Ringer’s solution (in mm:145 NaCl, 2.7 KCl, 1 MgSO_4_, 1.2 CaCl_2_, 1.55 Na_2_HPO_4_, 0.445 NaH_2_PO_4_, pH 7.3 at RT) was continuously pumped through the probe at 1.5 µl/min during the first 30–60 min after probe insertion. Then the pumping speed was reduced to 0.3 µl/min until the next day. To prevent the microdialysis probe, which was secured to the animals’ head, from being subjected to the torque created during the movement of animal, the rats was fitted with a custom-made harness, and the harness was attached to a swivel (liquid commutator 375/22 or 375/D/22, Instech Laboratories) by a flexible stainless steel cable. Rats were left overnight in the testing chamber with food and water *ad libitum*.

### Microdialysis

Sample collection was initiated the next morning, and all samples were collected in the light phase during 08:00–12:30. All dialysates were briefly stored on ice in the dark, and then manually injected into the HPLC-ECD system for measuring DA concentration in dialysates during 08:00–15:00 of the same day. Dialysate was collected into vials mounted just above the harness assembly. Drugs and hormones of interest were administered systemically (i.p. or s.c.) or intrastriatally via the microdialysis probe (reverse dialysis). For delivering E2, ICI, or MPEP via reverse dialysis method, drugs were first dissolved in pure UPS grade ethanol as 1000× (or above) stock solution; then, at use, they were further freshly diluted in Ringer’s solution and manually filtered via 0.2 μm syringe filters. With reverse dialysis, the drug of interest passes through the membrane of a microdialysis probe and diffuses into the striatum down a concentration gradient. Based on the *in vitro* results, we estimate that the efficiency of drug delivery with infusion method is 3–10% (data not shown). Thus, the effective concentration in the brain is considerably lower than the concentration in the probe. Thirty-sixty minutes before the first sample collection, the pumping speed was increased to 1.5 µl/min. Each dialysate sample was collected for 10 min. Baseline samples were collected for 30 min. When drugs were delivered via reverse dialysis, five samples were collected after the solutions were changed and the last three samples were used as the new baseline (it took ∼20 min for a new solution to reach equilibrium in the system). All rats in all experiments received an AMPH injection during microdialysis (2.5 mg/kg in saline, i.p.) and 10 min samples were collected for the following 2 h (12 samples).

### Treatment protocols for each experiment before AMPH administration

**Figure 1:**
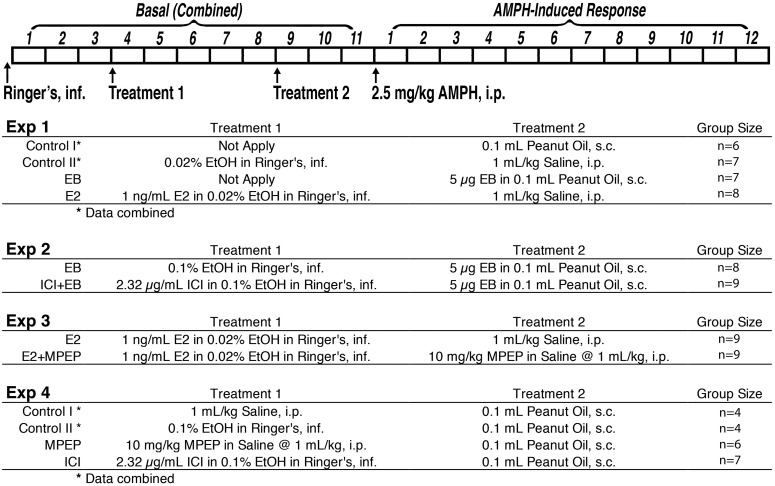
Schematic diagram of all the treatments in each experiment.

Figure 1 shows treatment details during the microdialysis sample collection in each experiment. Briefly, all rats were infused with Ringer’s solution for determining baseline DA and then treated with one or two pretreatments before AMPH injections. Specifically, in Experiment 1, rats were randomly assigned to 1 of 4 groups: (1) E2 Group (*n* = 7), rats were infused with Ringer’s solution with E2 in it (1 ng/ml E2; first dissolved in 100% ethanol and then diluted in Ringer’s solution, ethanol final concentration 0.02%); (2) Estradiol benzoate (EB) Group (*n* = 8), rats were treated with a subcutaneous injection of EB (5 µg in 0.1 ml peanut oil); (3–4) Control Groups, rats received either a subcutaneous injection of peanut oil (0.1 ml per rat; *n* = 6) or 0.02% ethanol in Ringer’s solution (vehicle for E2; *n* = 7). Rats that were treated with peanut oil or ethanol in Ringer’s solution did not significantly differ from each other and were combined in the analyses. There were two groups in Experiment 2: the ICI Group (*n* = 9) was infused with Ringer’s solution with ICI in it (2.32 µg/ml ICI, which is an equimolar concentration to E2 1 ng/ml; first dissolved in 100% ethanol and then diluted in Ringer’s solution; ethanol final concentration was 0.1%). The rats then received a subcutaneous EB injection following the ICI treatment. Control Group (*n* = 8) received Ringer’s solution with 0.1% ethanol (vehicle for ICI), followed by an EB administration. Experiment 3 also had two groups: E2 + MPEP Rats (*n* = 9) received E2 via reverse dialysis as described above and an intraperitoneal MPEP injection (10 mg/kg). Control rats (*n* = 9) received E2 via reverse dialysis and an intraperitoneal saline injection. In Experiment 4, rats were assigned into 1 of the 4 groups: ICI group (*n* = 7), where ICI dissolved in Ringer’s solution was administered via reverse dialysis; MPEP group (*n* = 6), where MPEP was injected systemically as above; and two control groups where rat received intraperitoneal saline (*n* = 4) or ICI vehicle (*n* = 4). The two control groups were combined because of similar levels of baseline DA as well as DA concentrations following AMPH injections. All rats were injected with AMPH following these pretreatments and dialysate samples from the DLS were collected every 10 min for 2 consecutive hours.

### DA concentration measurement by HPLC

DA concentration was assayed using a HPLC-ECD system described in (Hu and Becker, 2003). In brief, dialysate samples were separated on an ESA (ESA Biosciences) HPLC column (HR-80 × 3.2, 3 μm particle size, 80 mm length) at 40°C, with a mobile phase consisting of the following (in mm): 75 NaH_2_PO_4_, 0.2 EDTA, 1.4 OSA (1-octanesul fonic acid sodium salt monohydrate; Fluka, catalog #74882) and 17% methanol in HPLC water, pH 4.7). Flow rate through the column was set to 0.7 ml/min. Dopamine was quantified using a coulometric detector (Coulochem II, ESA) equipped with a high-sensitivity analytical cell containing dual coulometric working electrodes (ESA, model #5014B). The detector settings were as follows: Detector 1, −150 mV; Detector 2, +100 mV; and guard cell +300 mV. Output from Detector 2 was used for dopamine quantification. The retention time of DA was ∼2.5 min.

### Histology

Four to 7 d following completion of microdialysis, animals received an overdose of anesthesia and were sacrificed. Their brains were prepared for histologic analysis using standard techniques for frozen sections and cresyl violet staining was used to determine the location of the microdialysis probes. Only data from the rats where probes were located inside the DLS are reported here. Two rats were excluded because of the probes going too ventral and six more rats were also excluded because of probe damage or sickness.

### Statistical analyses

We used software SPSS V24 in all data analyses. Data were expressed in mean ± SEM. The percentage increase from baseline of each rat was used to assess DA response to AMPH in each 10 min sample. Baseline was determined by the mean of all samples before AMPH injections because no difference in DA concentrations was found in these samples (data not shown). Mixed-design repeated-measure ANOVA was used to examine treatment effect (e.g., ICI vs vehicle) among groups and the effect of time on DA concentrations within each group. We focused our analyses a priori on the first four samples collected following AMPH to catch patterns of peak DA concentrations in each condition. When significant effects of treatment were found, one-way ANOVA or *t* test was used to determine whether there was a significant difference in the each of the four samples post-AMPH among treatment group(s) and the control group. A priori planned contrast *post hoc* analysis was used to examine differences among >2 groups. Two data points in the first four samples post-AMPH of all rats (from 2 separate rats) were missing because of technical issues and were replaced by the average of the data points right before and after. In cases when assumptions for parametric tests were not met, nonparametric tests (e.g., Mann–Whitney *U* and Kruskal–Wallis tests) were used.

## Results

**Figure 2:**
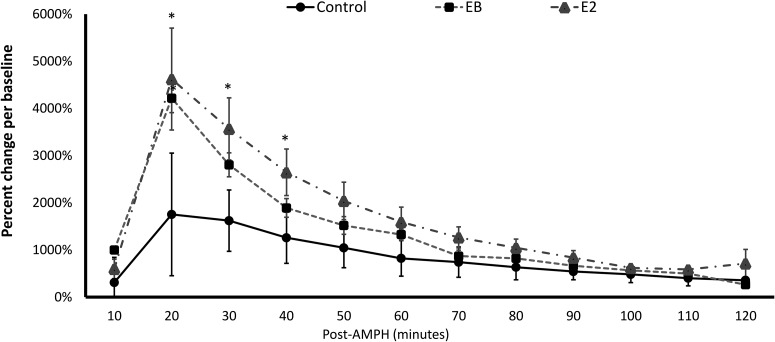
E2 in the DLS or EB, subcutaneously, potentiates DA release following AMPH injections. E2 was dissolved in Ringer’s solution and was infused into the DLS via reverse microdialysis. *indicates a significant difference between rats treated with E2 or EB and control rats.

Experiment 1: as can be seen in Figure 2, E2 delivered via reverse dialysis directly into the DLS or EB subcutaneously significantly enhanced AMPH-induced striatal DA release relative to the control group. Repeated-measures test showed there were a significant effect of treatment (*F*_(2,25)_ = 4.659, *p* = 0.019) and a significant interaction effect of Treatment × Time (*F*_(6,75)_ = 3.640, *p* = 0.003) in the DLS DA concentrations of the first four samples following AMPH injections. Planned *post hoc* comparison tests showed there were significant effects of E2 and EB compared with controls (*p* = 0.009 and *p* = 0.048, respectively). To better understand the time course on the differentiated elevation of peak DA levels among the three groups, one-way ANOVA tests (and a priori planned *post hoc* comparisons) were used to compare each of the four DA concentrations across conditions. Significant differences were found between the E2- and EB-treated rats and the control rats in the DA concentrations shortly after AMPH injections (Table 1).

**Table 1:** : Comparisons of DA release in response to AMPH among rats with varying pretreatments in Experiments 1–3

**ANOVA tests comparing rats treated with EB, E2, and Vehicle in Experiment 1**				
Time post-AMPH, min	10	20	30	40
*F* value	*F*_(2,25)_ = 1.853	*F*_(2,25)_ = 4.433	*F*_(2,25)_ = =5.045	*F*_(2,25)_ = 3.939
*p* value	0.178	**0.023**	**0.014**	**0.033**
**Planned contrast tests**				
Time post-AMPH, min	10	20	30	40
*p* Value, Control vs EB	NA	**0.037**	0.083	0.233
*p* Value, Control vs E2	NA	**0.013**	**0.005**	**0.010**
**Mann-Whitney *U* tests comparing rats treated with EB and ICI+EB in Experiment 2**				
Time post-AMPH, min	10	20	30	40
*U*	14.000	14.000	12.000	9.000
*p* value	**0.034**	**0.034**	**0.021**	**0.009**
**Independent tests comparing rats treated with E2 and E2+MPEP in Experiment 3**				
Time post-AMPH, min	10	20	30	40
*t* value	*t*_(16)_ = 1.410	*t*_(16)_ = 2.790	*t*_(16)_ = 2.232	*t*_(16)_ = 1.993
*p* value	0.178	**0.013**	**0.040**	0.064

Values in bold indicate significant differences.

**Figure 3:**
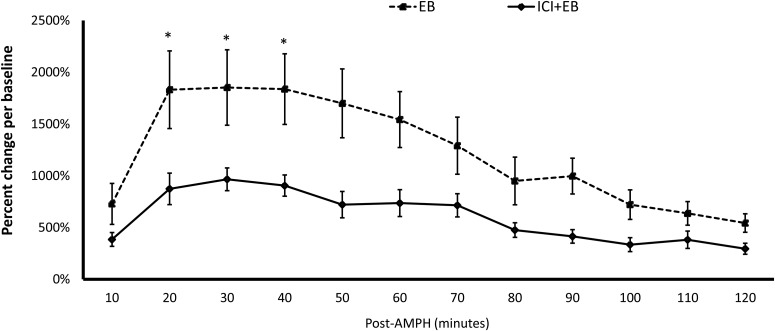
ICI infused into the DLS reduces E2-induced DA potentiation following AMPH injections. *indicates a significant difference between rats treated with ICI and control rats.

Experiment 2: as shown in Figure 3, ICI significantly decreased EB-induced enhancement in the DA release in the DLS after an intraperitoneal injection of AMPH. A significant main effect of Treatment was found in the measured DA concentrations in the DLS (*F*_(1,15)_ = 7.360, *p* = 0.016). There was also a significant interaction between Treatment × Time (repeated-measures: *F*_(3,45)_ = 4.045, *p* = 0.010). Mann–Whitney *U* tests showed all the four samples collected after right after AMPH injections differed in DA concentrations for ICI-treated versus vehicle-treated rats (assumptions for parametric *t* tests were not met, so nonparametric tests were used; Table 1).

**Figure 4:**
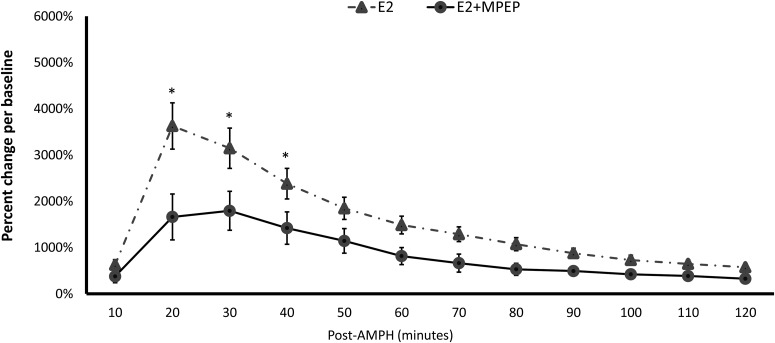
MPEP reduces E2-induced DA potentiation following AMPH injections. *indicates a significant difference between rats treated with MPEP and control rats.

Experiment 3: as shown in Figure 4, MPEP also significantly decreased EB-induced DA potentiation in the DLS post-AMPH treatment. There were a significant main effect of Treatment in the DA concentrations (repeated-measures: *F*_(1,16)_ = 5.895, *p* = 0.027) as well as a significant interaction of Treatment × Time in the DA concentrations (repeated-measures: *F*_(3,48)_ = 6.031, *p* = 0.001). Independent *t* tests showed in nearly all samples after AMPH injections there were significant differences between rats treated with MPEP versus those with saline in DA concentrations (Table 1).

**Figure 5:**
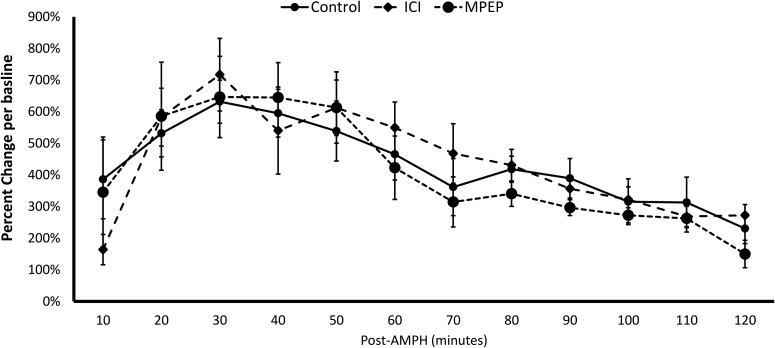
Neither ICI or MPEP influences DA release in the DLS when estradiol was not administered. These rats were ovariectomized and were not given EB injections or E2 infusions.

Experiment 4: as shown in Figure 5, MPEP or ICI did not influence DA release post-AMPH when E2 was not administered in OVX rats, unlike what was seen in Experiments 2 and 3. The increase in DA concentrations post-AMPH administration did not differ in MPEP or ICI-treated rats versus controls. There was a significant effect of Time (*F*_(3,45)_ = 16.300, *p* = 2.598E−7), but there was no main effect of Treatment (*F*_(2,15)_ = 0.140, *p* = 0.870) neither was there a significant interaction between Treatment and Time (*F*_(6,45)_ = 0.925, *p* = 0.487).

Lastly, there was no effect of EB, E2, ICI, or MPEP on the DA release in the DLS prior to AMPH injections across all experiments (data not shown). This lack of difference indicates the above agents do not affect DA release independent of AMPH. 

## Discussion

The present study showed E2 enhances DA release in the DLS following AMPH administration. This enhancing effect of E2 is mediated by E2 receptors and mGlu5 receptors as blocking E2 receptors in the DLS by ICI or intraperitoneal injections of mGlu5 receptor antagonist MPEP inhibits the E2-induced DA elevation in DLS. We also showed that ICI and MPEP are not able to influence DA levels in the DLS when E2 is not administered in OVX rats.

There is mounting evidence that E2 has been implicated in addictive behavior. E2 enhances ethanol reward in female mice (Hilderbrand and Lasek, 2018). E2 is even found to increase choice of cocaine over food in male rats as observed in females (Bagley et al., 2017). Our data support the enhancing effect of E2 on reward and thus the notion that it exacerbates addictive behavior, as it increases dopamine levels in response to AMPH challenge. Interestingly, there is considerable evidence that estradiol reduces food intake in female rats (Yu et al., 2008; Butera et al., 2010; Santollo et al., 2010; Santollo and Daniels, 2015; Butler et al., 2018; but see Boswell et al., 2006; Butera et al., 2010). The mechanisms underlying the apparent differences in the roles of E2 in motivated behaviors are less well understood, but it could be that E2 acts in different brain regions to modulate different types of rewards (e.g., drug addiction vs food reward).

The ability of E2 in influencing addiction or reward may be because of its action in the midbrain dopamine reward system. Mice treated with E2 or ERβ agonists showed increased conditioned place preference for cocaine, whereas specific knockdown of the ERβ gene decreased cocaine conditioned place preference (Satta et al., 2018). Another study shows E2 acts on ventral tegmental area to increase the sensitivity of dopamine neurons to ethanol (Vandegrift et al., 2017). E2 in the MPOA also increases DA release in the NAc in response to cocaine (Tobiansky et al., 2016). Our finding showed that E2 in the DLS potentiates dopamine release following AMPH injections. DLS plays a critical role in addictive behavior in both rodent and human studies. In humans, damage to dorsal striatum alleviates addiction to alcohol and nicotine (Muskens et al., 2012). In rodent studies, it has been suggested that dorsal medial striatum and NAc are crucial in the initial acquisition of the reward and then DLS and NAc begin to take over when the behavior becomes more addiction-like. Together, it is possible that E2 acts on different regions to convergently modulate addictive behavior.

Both ERα and ERβ have been reported in the E2 modulation of addictive behavior. The ERα agonist (propyl-pyrazole triol) and the ERβ agonist (diarylpropionitrile), independently increased choice on the high-reward tested in an operant chamber (Uban et al., 2012). These effects were most pronounced 24 h after administration suggesting genomic action of the receptors. Effects of E2 via its action on membrane receptors have been debated (Govind and Thampan, 2003) and there is increasing evidence showing rapid effects of E2 that are likely via non-genomic receptors (Revankar et al., 2005; Micevych et al., 2015; Paletta et al., 2018; Yoest et al., 2018). E2 is found to exert its effects via acting on G-protein-coupled estrogen receptors (GPER-1), as well as ERα and ERβ receptors, to rapidly facilitate short-term memory in female mice (Lymer et al., 2018). Our finding in the present study showed that E2 rapidly potentates dopamine release following AMPH treatment in the DLS. It will be important to further investigate the roles of each receptor type/location in these effects.

Several studies have shown that mGlu5 is involved in the effects of E2 in the regulation of behavior and physiology (Grove-Strawser et al., 2010; Peterson et al., 2015; Al-Sweidi et al., 2016). E2 is reported to mediate dendritic spine plasticity in the NAc through activation of mGlu5, evaluated via DiI labeling and confocal microscopy (Peterson et al., 2015). The results of Peterson et al. (2015) suggest E2’s role in mediating neuronal plasticity in the NAc via mGlu5 is important for E2’s effect in drug addiction. Another study shows E2 facilitates cocaine self-administration in OVX rats and mGlu5 activation is essential for this effect (Martinez et al., 2016). The study also demonstrates direct activation of mGlu5 is insufficient to mimic the effect of E2 in cocaine self-administration, suggesting that E2 receptors possibly need to be activated simultaneously to have the effect. Together, these findings are consistent with the results of the present study that both E2 receptors and mGlu5 s are necessary for E2’s potentiation in DA release in DLS. It will be important to extend these results by examining the involvement of mGlu5 in other E2-mediated behaviors.

Although it is clear that both E2 receptors and mGlu5 are required for the estradiol evoked DA release from the DA terminals, our study does not show whether estradiol directly acts on or whether the two receptors are on the DA neurons. In fact, studies suggest estradiol activates E2 receptors coupled with mGlu5S on MSNs, which then modulates the release of GABA to influence DA terminals (Schultz et al., 2009). E2 receptors can be anchored to plasma membrane via caveolin protein, which then allow them to functionally couple with mGluRs (Yoest et al., 2018). The authors propose that E2 and mGlu receptors collaboratively act on MSNs in the DLS to modulate DA release from DA neuronal terminals. It is also possible that E2 acts on other interneurons (such as cholinergic neurons) to modulate DA release in the DLS, or influences glutamate release on cortical afferents.

Our data demonstrated marked increase of DA release in DLS following AMPH injections. This effect has been reported both *in vivo* and *in vitro* in our previous studies (Becker and Ramirez, 1981; Xiao and Becker, 1998; Becker and Rudick, 1999). Because of unknown vendor/batch effects, different magnitudes of overall increase in DA concentrations following AMPH administration were observed in Experiments 1 and 3 (rats from Harlan Laboratories) than in Experiments 2 and 4 (rats from Charles River Laboratories).

## Conclusion

The present study demonstrates that E2 directly potentiates the AMPH-induced increase in DA in the DLS. The effects of E2 are mediated by E2 receptors and can be blocked by an mGlu5 antagonist. These results provide important information on the neural mechanism through which E2 may contribute to sex differences in behaviors such as, but not limited to, addictive behavior. Our data also suggest that targeting mGlu receptors could be a potential treatment for E2 related disorders in female individuals.

## Synthesis

Reviewing Editor: Karen Szumlinski, University of California at Santa Barbara Decisions are customarily a result of the Reviewing Editor and the peer reviewers coming together and discussing their recommendations until a consensus is reached. When revisions are invited, a fact-based synthesis statement explaining their decision and outlining what is needed to prepare a revision will be listed below. The following reviewer(s) agreed to reveal their identity: Robert Meisel, Erin Calipari.

Upon consultation with two reviewers with expertise in neurohormone regulation of addiction-related behavior, I am pleased to consider your paper for acceptance in eNeuro, pending revisions. Both reviewers and I considered this work to be novel and we all agreed that the findings significantly advance our knowledge pertaining to mGlu5-estradiol interactions in regulating dopamine release within striatum. Reviewer 1 provides some suggestions to steam-line the report and requests some relatively minor revisions pertaining to methodological details that are readily addressable. Reviewer 2 highlights some inconsistency in terms of the proposed mechanism(s) for the effects on dopamine release and all three reviewers agree that a more thorough discussion regarding potential indirect mechanism(s) would improve the report. I ask that the authors please correct “mGluR5" to the newer nomenclature “mGlu5" throughout. As these revisions should require less than 2 months' work, we look forward to reviewing a revised report in the near future. Please find below the specific comments of each reviewer to facilitate your revision.

Reviewer 1:

This is a nicely written manuscript describing the results of a study designed to examine mechanisms underlying the effects of estradiol in the dorsal striatum on amphetamine-stimulated dopamine release. The study is nicely designed and comprehensive. I have only a few minor comments on the manuscript.

1) There is no indication of postsurgical analgesia or antibiotic treatments. Is this an oversight in the manuscript or were no treatments given?

2) The issue of mechanisms of estradiol effects on feeding (p. 13) is far too complicated for discussion here and goes beyond the scope of the manuscript. I recommend deleting this paragraph

3) There is discussion of GPER-1 possibly mediating the effects of estradiol on dopamine release. Does the ICI compound block GPER-1 receptors? If not, the data would suggest this receptor is not involved in the reported enhancement of dopamine release.

4)The last paragraph of the discussion (p. 15)should be worded in a more positive way emphasizing the conclusion that despite the variability the results maintain their validity. The vendor issues are a distractor as the authors have not identified this as the key variable underlying the inter-experiment differences.

5) Figure 6 is not needed and can just be referred to as data not shown.

Reviewer 2

Comments to the Authors (Required) The manuscript outlines the effects of estradiol (E2) on dopamine overflow in dorsal striatal regions. This is a timely and important study to understand how E2 is affecting dopamine circuit regulation. The study is well designed, and the experiments are well executed - however, it will be important for the authors to refine the discussion to more accurately outline the system and how these effects could be taking place. While it is clear that E2 and mGlur5 both are able to regulate evoked dopamine release, some of the proposed mechanisms are unclear and the discussion could benefit from a more through outline of how these potential effects could be taking place.

At some places the authors seem to loosely hypothesize is that E2 could be acting directly on dopamine terminals; however, with this technique it is impossible to tell if that is the case - and, in fact the data suggests against that. The authors do state later that their hypothesis is that it acts on MSNs. The authors should be careful with some of the wording in this regard.

The authors hypothesize that E2 acting on MSNs could act to modulate dopamine release; however, it is not clear what the local circuit regulation would be in this case? Do the authors think that GABA release from the MSNs regulates dopamine terminals directly - through GABA-B receptors? Or differences in neuromodulatory systems such as dynorphin acting on Kappa receptors on the presynaptic terminal?

mGlur5 is also located on glutamatergic inputs into the NAc. Do the authors think that their action at glutamatergic inputs could play a role in this process?

It is also possible that the E2 is modulating cholinergic interneurons that have been shown to regulate dopamine release. Glutamatergic inputs into the NAc that synapse on ChAT positive interneurons are able to evoke dopamine release. Thus, It is possible that E2 is regulating cholinergic excitability, which would be a potential mechanisms by which dopamine release could be modulated. This would be a potential mechanism that could be discussed.

## Author Response

We appreciate the thoughtful comments and helpful suggestions from all three reviewers.

Synthesis Statement for Author (Required): Upon consultation with two reviewers with expertise in neurohormone regulation of addiction-related behavior, I am pleased to consider your paper for acceptance in eNeuro, pending revisions. Both reviewers and I considered this work to be novel and we all agreed that the findings significantly advance our knowledge pertaining to mGlu5-estradiol interactions in regulating dopamine release within striatum. Reviewer 1 provides some suggestions to steam-line the report and requests some relatively minor revisions pertaining to methodological details that are readily addressable. Reviewer 2 highlights some inconsistency in terms of the proposed mechanism(s) for the effects on dopamine release and all three reviewers agree that a more thorough discussion regarding potential indirect mechanism(s) would improve the report. I ask that the authors please correct “mGluR5" to the newer nomenclature “mGlu5" throughout. As these revisions should require less than 2 months' work, we look forward to reviewing a revised report in the near future. Please find below the specific comments of each reviewer to facilitate your revision. Thanks. “mGluR5" has now been changed to “mGlu5". All the changes have been highlighted in yellow background in the manuscript.

Reviewer 1: This is a nicely written manuscript describing the results of a study designed to examine mechanisms underlying the effects of estradiol in the dorsal striatum on amphetaminestimulated dopamine release. The study is nicely designed and comprehensive. I have only a few minor comments on the manuscript.

1) There is no indication of postsurgical analgesia or antibiotic treatments. Is this an oversight in the manuscript or were no treatments given? We apologize for this oversight. Carprofen (5mg/Kg, subcutaneous injection) was administered daily for 3 days after surgery and triple antibiotic was given when necessary upon observation. Rats were monitored daily for 10 consecutive days for their recovery. The following sentences have been added to the end of the first paragraph on page 6: “Starting one day after the surgery (both cannula implantation and OVX), rats were administered with carprofen (5 mg/kg, s.c) daily for 3 consecutive days and triple antibiotic was given when necessary upon observation. All rats were observed at least once daily for 10 consecutive days to ensure their recovery."

2) The issue of mechanisms of estradiol effects on feeding (p. 13) is far too complicated for discussion here and goes beyond the scope of the manuscript. I recommend deleting this paragraph.

2

Thank you. The aim of that paragraph was to inform readers of the complexity of the possible mechanism of estradiol effects in distinct motivated behaviors, not to illustrate the mechanism of estradiol in regulating feeding behavior. The sentence 'Interestingly, activation of ERαs and GPER-1s, rapidly decreases food intake in female rats' in the manuscript has been changed to the following. 'Interestingly, there is considerable evidence that estradiol reduces food intake in female rats (Yu, Geary et al. 2008, Butera, Wojcik et al. 2010, Santollo, Katzenellenbogen et al. 2010, Santollo and Daniels 2015, Butler, Hildebrandt et al. 2018) (but see (Boswell, Reid et al. 2006, Butera, Wojcik et al. 2010)).'

3) There is discussion of GPER-1 possibly mediating the effects of estradiol on dopamine release. Does the ICI compound block GPER-1 receptors? If not, the data would suggest this receptor is not involved in the reported enhancement of dopamine release. That is an excellent question. There is not much data on the binding affinity of ICI to GPER1 receptors, but it is clear that ICI is an agonist at GPER-1 receptors in arcuate nucleus of the hypothalamus (Long et al., 2017, Hormones and Behavior). But we are currently investigating the effects of GPER-1 receptors in the rat striatum using the specific GPER-1 agonist, G1.

4) The last paragraph of the discussion (p. 15) should be worded in a more positive way emphasizing the conclusion that despite the variability the results maintain their validity. The vendor issues are a distractor as the authors have not identified this as the key variable underlying the inter-experiment differences. Thanks. The original sentences “There seems to have rat batch/vendor effects on the magnitudes of increase following AMPH administration in the present study, a greater overall increase in DA concentrations was observed in Experiments 1&3 (rats were from Harlan and testing was conducted at an earlier time before we switched to Charles Rivers) than in Experiments 2&4 (rats were from Charles River). We believe our conclusions from each experiment should still stand as they each had its own controls." are now changed to “Due to unknown vendor/batch effects, different magnitudes of overall increase in DA concentrations following AMPH administration were observed in Experiments 1&3 (rats from Harlan) than in Experiments 2&4 (rats from Charles River)."

5) Figure 6 is not needed and can just be referred to as data not shown. Ok, deleted.

Reviewer 2

Comments to the Authors (Required) The manuscript outlines the effects of estradiol (E2) on dopamine overflow in dorsal striatal regions. This is a timely and important study to

3

understand how E2 is affecting dopamine circuit regulation. The study is well designed, and the experiments are well executed - however, it will be important for the authors to refine the discussion to more accurately outline the system and how these effects could be taking place. While it is clear that E2 and mGlur5 both are able to regulate evoked dopamine release, some of the proposed mechanisms are unclear and the discussion could benefit from a more through outline of how these potential effects could be taking place. Thank you for the helpful comments. We added the following paragraph on Page 15 in the Discussion. 'While it is clear that both E2 receptors and mGlu5 are required for the estradiol evoked DA release from the DA terminals, our study does not show whether or not estradiol directly acts on or if the two receptors are on the DA neurons. In fact, studies suggest estradiol may activate E2 receptors coupled with mGlu5S on MSNs, which then modulates the release of GABA to influence DA terminals (Schultz, von Esenwein et al. 2009). E2 receptors can be anchored to plasma membrane via caveolin protein which then allows them to functionally couple with mGluRs (Yoest, Quigley et al. 2018). The authors propose that E2 and mGlu receptors collaboratively act on MSNs in the DLS to modulate DA release from DA neuronal terminals. It is also possible that E2 acts on other interneurons (such as cholinergic neurons) to modulate DA release in the DLS.' Also, we modified the following sentence in the Introduction. 'In dorsal striatum (DS), a region that is critical for habitual drug taking behavior, E2 modulates behavior by acting on medium spiny neurons (Mermelstein, Becker et al. 1996) and by altering DA transmission (Xiao and Becker 1994, Xiao and Becker 1998). ' to 'In dorsal striatum (DS), a region that is critical for habitual drug taking behavior, E2 modulates behavior by acting on GABA medium spiny neurons (Mermelstein, Becker et al. 1996) and by altering DA transmission indirectly through a presynaptic mechanism (Xiao and Becker 1994, Xiao and Becker 1998).

At some places the authors seem to loosely hypothesize is that E2 could be acting directly on dopamine terminals; however, with this technique it is impossible to tell if that is the case - and, in fact the data suggests against that. The authors do state later that their hypothesis is that it acts on MSNs. The authors should be careful with some of the wording in this regard. Agreed. With the present study we can't tell one way or the other --- whether or not E2 acts directly on DA terminals. See our response to the above comment.

The authors hypothesize that E2 acting on MSNs could act to modulate dopamine release; however, it is not clear what the local circuit regulation would be in this case? Do the authors think that GABA release from the MSNs regulates dopamine terminals directly -

4

through GABA-B receptors? Or differences in neuromodulatory systems such as dynorphin acting on Kappa receptors on the presynaptic terminal? Again, in the present study we do not have direct evidence to show one way or the other. Other studies have suggested that GABA from MSNs presynaptically regulates DA release (see Yoest et al., 2018 and Schultz et al., 2009). Whether the MSNs that are modulated by E2 are co-releasing dynorphin to contribute to the effects observed is beyond the scope of this study.

mGlu5 is also located on glutamatergic inputs into the NAc. Do the authors think that their action at glutamatergic inputs could play a role in this process? We do not know whether modulation of glutamate release by mGlu5 is involved with the results reported here.

It is also possible that the E2 is modulating cholinergic interneurons that have been shown to regulate dopamine release. Glutamatergic inputs into the NAc that synapse on ChAT positive interneurons are able to evoke dopamine release. Thus, It is possible that E2 is regulating cholinergic excitability, which would be a potential mechanisms by which dopamine release could be modulated. This would be a potential mechanism that could be discussed. Thanks. E2 also increases DA levels in the NAc and it is indeed possible that cholinergic interneurons play a role in this E2 induced effect. So E2 could do the same thing in the DS as in the NAc (there are cholinergic neurons in the DL). We have added this in the new paragraph in the discussion. See our response to point 1 of this reviewer.
